# Improving the Charge Carrier Transport and Suppressing Recombination of Soluble Squaraine-Based Solar Cells via Parallel-Like Structure

**DOI:** 10.3390/ma11050759

**Published:** 2018-05-09

**Authors:** Youqin Zhu, Jingli Liu, Jiao Zhao, Yang Li, Bo Qiao, Dandan Song, Yan Huang, Zheng Xu, Suling Zhao, Xurong Xu

**Affiliations:** 1 Key Laboratory of Luminescence and Optical Information of Ministry of Education, Beijing Jiaotong University, Beijing 100044, China; 14121634@bjtu.edu.cn (Y.Z.); 15121568@bjtu.edu.cn (J.L.); 14118436@bjtu.edu.cn (J.Z.); bjliyang2013@163.com (Y.L.); boqiao@bjtu.edu.cn (B.Q.); ddsong@bjtu.edu.cn (D.S.); zhengxu@bjtu.edu.cn (Z.X.); xrxu@bjtu.edu.cn (X.X.); 2 Institute of Optoelectronics Technology, Beijing Jiaotong University, Beijing 100044, China; 3 Key Laboratory of Green Chemistry and Technology of Ministry of Education, College of Chemistry, Sichuan University, Chengdu 610064, China

**Keywords:** small molecule organic solar cell, squaraine, ternary, charge carrier mobility, parallel-like, TPV

## Abstract

Small molecule organic solar cells (SMOSCs) have attracted extensive attention in recent years. Squaraine (SQ) is a kind of small molecule material for potential use in high-efficiency devices, because of its high extinction coefficient and low-cost synthesis. However, the charge carrier mobility of SQ-based film is much lower than other effective materials, which leads to the pretty low fill factor (FF). In this study, we improve the performance of SQ derivative-based solar cells by incorporating PCDTBT into LQ-51/PC_71_BM host binary blend film. The incorporation of PCDTBT can not only increase the photon harvesting, but also provide an additional hole transport pathway. Through the charge carrier mobility and transient photovoltage measurement, we find that the hole mobility and charge carrier lifetime increase in the ternary system. Also, we carefully demonstrate that the charge carrier transport follows a parallel-like behavior.

## 1. Introduction

Organic solar cells (OSCs) are a candidate renewable energy conversion device, due to their advantages of being low-cost, lightweight, having a large area, and being flexible, which makes them amenable to roll-to-roll high volume production [1,2,3,4]. The power conversion efficiency (PCE) has exceeded 13% in recent years, on account of the combination of novel materials synthesis, device engineering, optimization of progress control, and physical mechanism research [5,6,7]. Although the best performance of organic solar cells is achieved by polymer donor/nonfullerene acceptor systems, small molecule donors are alternatives due to their advantages of easier synthesis and purification than polymers [8,9]. The squaraine (SQ) dyes have high absorption efficiency (~10^5^ M^−1^ cm^−1^), and it is easy to tailor the bandgap by molecular design, photochemical and thermal stability, and low-cost synthesis, which makes them good candidates for donor materials [10,11,12,13]. Nevertheless, the charge carrier mobility of SQ is relatively low (~10^−5^ cm^2^/Vs) [14,15], limiting the short circuit current (J_SC_) and FF of the device based on SQ dyes.

Most SQ dyes are amorphous in the blend films, and cannot form a continuous charge carrier transport pathway, which leads to inefficient charge carrier transport and significant recombination in SQ-based solar cells. Some processing treatments, such as solvent additive, thermal annealing, and solvent vapor annealing, are beneficial to improving the morphology of blend films [16,17,18]. Through recent efforts, the PCE of solution processing SQ-based solar cells is more than 7% [12,13]. However, there are some SQ derivatives insensitive to these processing treatments [14]. Also, the absorption spectrum of organic materials is still not broad enough to harvest most photons. These factors limit the further improvement of the device performance.

In this article, we incorporate wide-bandgap polymer poly[[9-(1-octylnonyl)-9H-carbazole-2,7-diyl]-2,5-thiophenediyl-2,1,3-benzothiadiazole-4,7-diyl-2,5-thiophenediyl] (PCDTBT) into narrow-bandgap SQ derivative 4-((3-benzyl-1,1-dimethyl-1H-benzo[e]indol-3-ium-2-yl)methylene)-2-(2,6-dihydroxy-4-(1,3,3a,8b-tetrahydrocyclopenta[b]indol-4(2H)-yl)phenyl)-3-oxocyclobut-1-enolate (LQ-51)/[6,6]-phenyl C_71_ butyric acid methyl ester (PC_71_BM) small molecule binary host film to increase the photon harvesting and hole mobility of blend films. The charge carrier lifetime increases in solar cells incorporated with PCDTBT, which results from the additional hole transport pathway and the high hole mobility of PCDTBT. In addition, the charge carrier transport is carefully demonstrated following parallel-like behavior. As a result, the V_OC_ J_SC_, FF, and PCE of SQ derivative-based solar cells simultaneously increase.

## 2. Materials and Methods

The asymmetric squaraine derivative LQ-51 was synthesized by ourselves. The PCDTBT and PC_71_BM were bought from 1 Material Inc (1 Material, Dorval, QC, Canada), and used as received. LQ-51 and PC_71_BM were dissolved in chloroform (CF) with a weight ratio of 1:5, and the total concentration of 18 mg/mL, named solution A. Solution B contained LQ-51, PCDTBT, and PC_71_BM with a weight ratio of 1:1:5, and total concentration of 21 mg/mL dissolved in CF. The solutions A and B were stirred in an N_2_-filling glove box at 25 °C for 12 h. Then, solution A and B were mixed in proportional volumes to make solutions of LQ-51/PCDTBT/PC_71_BM with a weight ratio of 1:X:5 (X = 0, 0.1, 0.3, 0.5, 0.7, and 1), which were stirred for 4 h before spin-coating on the substrates.

Patterned indium tin oxide (ITO) glass substrates with a sheet resistance of 15 Ω sq^−1^ were cleaned in soap deionized water, deionized water, and anhydrous ethanol for 25 min at each step and finally blow-dried by nitrogen. After ultraviolet/ozone treatment for 5 min, 80 Å MoO_3_ as the hole transport layer (HTL) was thermally evaporated onto the ITO glass substrates at a speed of 0.4 Å/s under a vacuum of 3 × 10^−4^ Pa. The active blend layers LQ-51/PCDTBT/PC_71_BM = 1:X:5 were spin-coated onto the MoO_3_ layer at 2000 rpm for 50 s, and completely dried in covered glass Petri dishes for 1 h in an N_2_-filled glovebox. Then, Poly [(9,9-bis(3′-(N,N-dimethylamino)propyl)-2,7-fluorene)-alt-2,7-(9,9–dioctylfluorene)] (PFN) as an electron transport layer (ETL) was spin-coated onto the active layer at 1500 rpm for 40 s, and completely dried in Petri dishes for 30 min in the glovebox. Finally, 100 nm Al as the cathode was deposited on the top at a speed of 2 Å/s under a vacuum of 3 × 10^−4^ Pa. The deposition speed and film thickness were monitored in situ by a quartz crystal oscillator mounted to the substrate holder. The active layer was 4 mm^2^.

The J–V characteristics of devices were measured by a Keithley 4200 source measurement unit (Cleveland, OH, USA) under AM 1.5 G illumination (100 mW cm^−2^). The absorption spectra of blend films were measured by a Shimadzu UV-3101 PC spectrometer (Shimadzu, Kyoto, Japan). The external quantum efficiency (EQE) curves were obtained using a QE/IPCE Measurements Solar Cell Scan 100 (Zolix, Beijing, China) system. Transient photovoltage (TPV) measurement was carried out as shown in the literature [19]. The solar cells were kept at open-circuit condition during TPV measurement by setting the input impedance of oscilloscope (Tektronix DPO 4104, Beaverton, OR, USA) at 1 MΩ. The white background light was provided by a Xenon Arc Light Source (Zolix LSH-X150, Zolix, Beijing, China) to keep the charge carrier density in device effectively constant. A 403 nm pulse laser as a small perturbation with frequency and pulse duration of 10 Hz and 100 μs, respectively, was driven by laser controller (ADR-1805, SFOLT, Shanghai, China) and pulse generator (Agilent 8114A, Agilent, Santa Clara, CA, USA) to generate a ΔV_OC_ around 5 mV, where ΔV_OC_ was the change of V_OC_ of the solar cell. The output voltage signal of the solar cell was detected by the digital oscilloscope. The charge carrier lifetime τ was determined by fitting the TPV signal with single-exponential decays. The distance between xenon arc light and the solar cell was changed to measure the charge carrier lifetime under different V_OC_ conditions.

## 3. Results and Discussion

**Figure 1:**
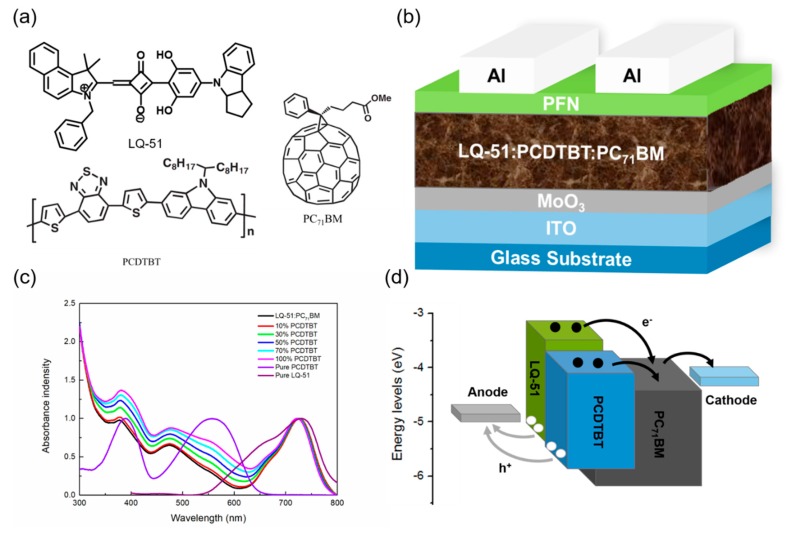
Material properties and device structure. (**a**) Chemical structures of LQ-51, PCDTBT, and PC_71_BM used in here; (**b**) Device architecture of organic solar cells; (**c**) The absorption spectra of binary and ternary films with different PCDTBT incorporation concentration; (**d**) The energy level diagram and proposed charge carrier transport paths in the device.

The chemical structures of the materials used in this work are as shown in Figure 1a, and the ternary device architecture used in this work is as shown in Figure 1b. The lowest unoccupied molecular orbital (LUMO) and the highest occupied molecular orbital (HOMO) energy levels are −3.40 and −5.10, −3.60 and −5.50, −4.30 and −6.10 eV, for LQ-51, PCDTBT, and PC_71_BM, respectively. The energy levels of PCDTBT are between LQ-51 and PC_71_BM, which can form a cascade energy structure when PCDTBT is incorporated. The absorption spectra of LQ-51, PCDTBT, and PC_71_BM neat films are as shown in Figure 1c. PCDTBT shows an absorption spectrum in the range from 350 nm to 650 nm, while, LQ-51 can harvest photons from 550 nm to 800 nm. The less overlapped absorption spectra, combined with cascade energy structure, make PCDTBT a suitable candidate in LQ-51/PC_71_BM ternary systems. The absorption spectra of binary and ternary films with different incorporated PCDTBT concentrations (10%, 30%, 50%, 70%, and 100%) are as shown in Figure 1c, which indicates that the incorporation of PCDTBT can enhance the photon harvesting. We also note that the absorption peak of LQ-51 is slightly blue-shifted when PCDTBT is incorporated, and the blue-shift extent increases as the incorporation concentration increases, which may be attributed to that PCDTBT disrupts the aggregation of LQ-51 and weakens the molecular interaction of LQ-51. The energy level diagram and proposed charge carrier transport paths in the device are as shown in Figure 1d.

**Figure 2:**
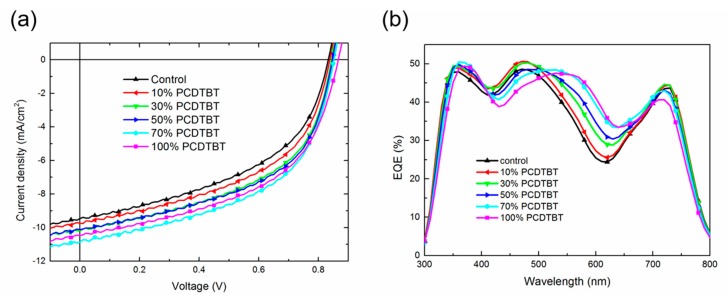
Device performance. (**a**) The current density–voltage (J–V) characteristics of binary and ternary devices; (**b**) The external quantum efficiency (EQE) curves of devices.

**Figure 3:**
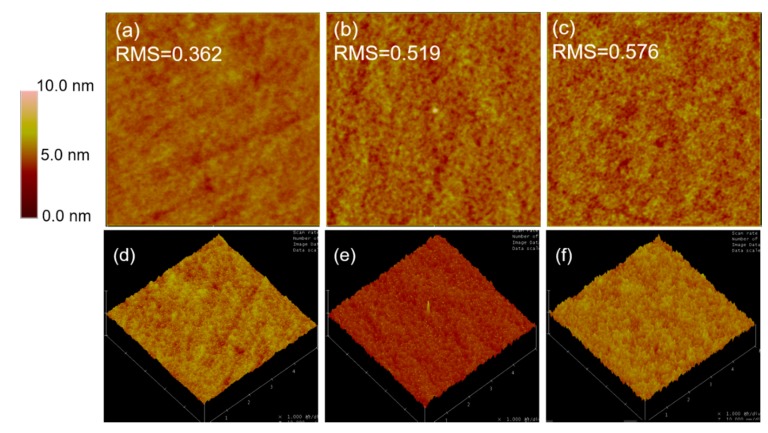
The atomic force microscope (AFM) height images and 3D images of (**a**,**d**) control binary film; (**b**,**e**) ternary blend film with 70% PCDTBT; (**c**,**f**) ternary blend film with 100% PCDTBT. The scan size of all images is 5 × 5 μm.

The current density–voltage (J–V) characteristics and photovoltaic parameters of binary and LQ-51/PCDTBT/PC_71_BM ternary solar cells are as shown in Figure 2a and Table 1, respectively. The open-circuit voltage (V_OC_), J_SC_, and FF of ternary solar cells increase monotonously compared with LQ-51/PC_71_BM binary solar cell when the incorporation concentration of PCDTBT increases up to 70%, from 0.83 V to 0.85 V, from 9.47 mA cm^−2^ to 10.86 mA cm^−2^, and from 47.77% to 51.98%, respectively, which results in a 28% increase of PCE from 3.75% to 4.80%. The V_OC_ increases to 0.87 V, while the J_SC_, FF, and PCE decrease slightly when the incorporation concentration increases continuously to 100%. The trends in series resistance (R_S_) and shunt resistance (R_Sh_) indicate the better FF in ternary devices. The R_S_ is a sum of the bulk resistance of various layers in devices and the contact resistance between them, while, the R_Sh_ is related to the quality of the films, such as traps and pinholes. The R_S_ decreases as the concentration of PCDTBT increases until the optimal ratio is reached, which results from the enhanced hole mobility of ternary films. While further increasing the ratio of PCDTBT to 100% leads to a larger R_S_, which may be attributed to a larger roughness of 100% PCDTBT ternary film and unefficient contact between blend film and modified electrodes. The atomic force microscope (AFM) images of blend films are as shown in Figure 3. The root-mean-square (RMS) roughness of control binary blend film, 70% PCDTBT incorporated ternary film, and 100% PCDTBT incorporated ternary film is 0.362 nm, 0.519 nm, and 0.576 nm, respectively. The R_Sh_ increases as the concentration of PCDTBT increases (from 321.54 Ω cm^2^ for the binary device to 555.56 Ω cm^2^ for 70% PCDTBT ternary device), which can be related to high-quality ternary films and less charge carrier recombination in ternary devices. We note that the binary solar cells fabricated with PCDTBT/PC_71_BM shows a much lower PCE (~3.4%) than the literature results (~6%, made from dichlorobenzene or chlorobenzene) [20,21,22], mainly because of adopting different donor/acceptor ratio (1:5) and solvent (CF) in this work. The performance of LQ-51/PC_71_BM solar cells made from CF is much better than that of devices made from dichlorobenzene and chlorobenzene (PCE lower than 2%). In consideration of the goal of the work, which mainly focuses on enhancing the performance of LQ-51/PC_71_BM system by parallel-like structure, we therefore did not use the optimum conditions for fabricating PCDTBT/PC_71_BM devices in this work.

The external quantum efficiency (EQE) curves of devices are as shown in Figure 2b. In the range of 350–400 nm and 540–650 nm, the EQE increases monotonously as the incorporation concentration of PCDTBT increasing, which can be accredited to the improved photon harvesting by PCDTBT, while the rise in EQE is smaller than that in absorbance intensity, which can be attributed to less efficient electron transport pathway in ternary films. In the range of 300–350 nm and 400–540 nm, the EQE increases when the incorporation concentration is 10% and 30%, then, it decreases when the concentration of PCDTBT further increases up to 50%. The photocurrent in these ranges mainly comes from the absorption of PC_71_BM, and slightly from PCDTBT. The large amount of incorporation of PCDTBT would disrupt the aggregation of PC_71_BM and destroy the electron transport path, which will lead to lower EQE, and will be further demonstrated by the charge carrier mobility measurement in the following. The EQE in the range of 660–800 nm is related to LQ-51, which decreases slightly when the incorporation concentration of PCDTBT is 70%, and decreases dramatically when the percentage is up to 100%. The significant decrease in this range results from the weak molecular interaction between LQ-51, which will hinder hole transport in LQ-51, and lead to a lower EQE. As a result, the optimal incorporation percentage of PCDTBT is 70%, where the ternary device can enhance photon harvesting and maintain efficient charge carrier transport simultaneously.

The changeless EQE in the response range of LQ-51 even when the incorporation concentration of PCDTBT up to 70% indicates that the ternary devices may work as a parallel-like structure [23], where the excitons generated in each donor material would diffuse to respective donor/acceptor interface, and dissociate into free electrons and holes. In this case, the electrons would transport through acceptor domain toward the cathode, while the holes would transport to the anode through the parallel pathways formed by two donors without charge transfer between two donors. The proposed charge transport pathways in blend films are as shown in Figure 1d.

**Figure 4:**
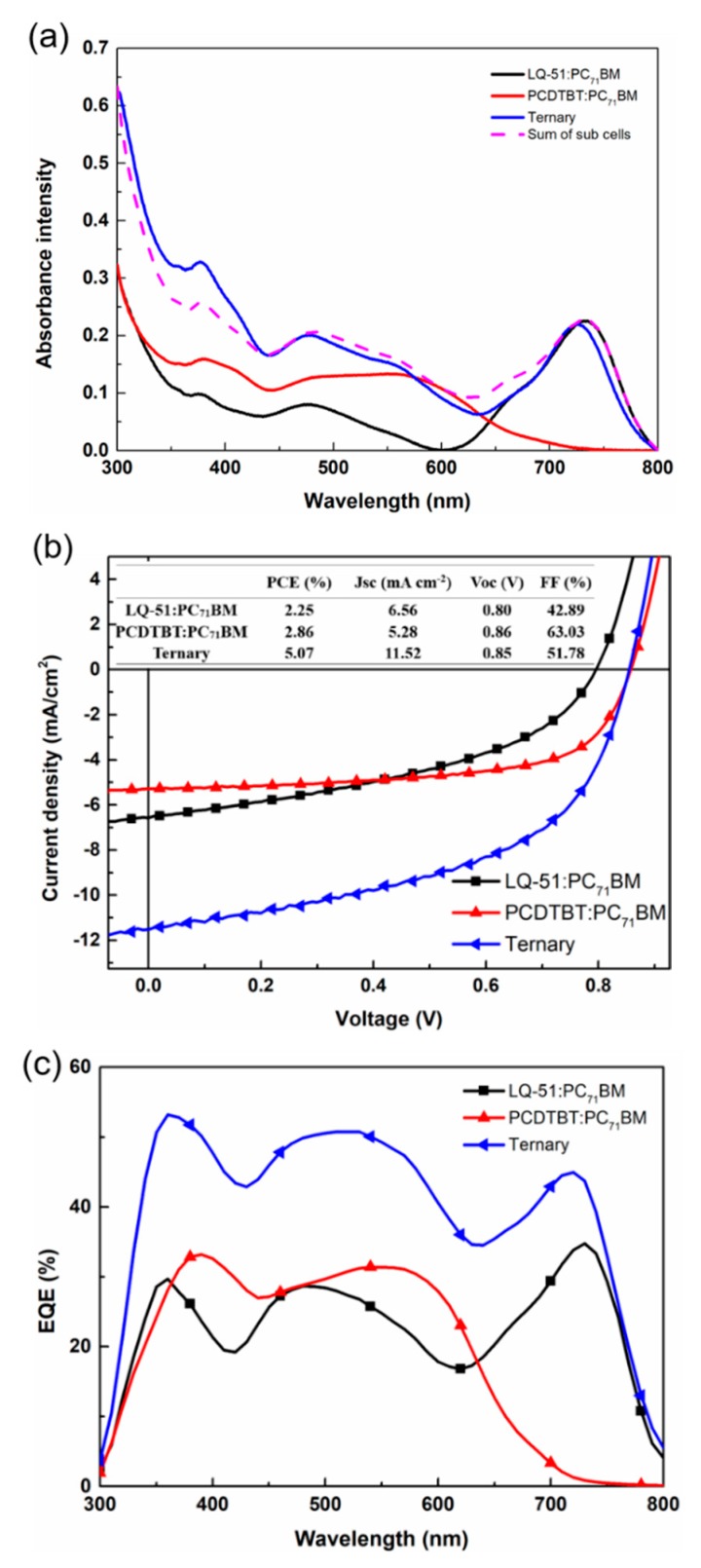
Properties of the ternary device and “sub-cells”. (**a**) The absorption spectra of LQ-51/PCDTBT/PC_71_BM (~117 nm), LQ-51/PC_71_BM (~60 nm), and PCDTBT/PC_71_BM (~55 nm) blend films; (**b**) The J–V characteristics of the ternary device and “sub-cells”; (**c**) EQE curves of the ternary device and “sub-cells”.

In order to demonstrate the parallel-like structure concept, we fabricated a ternary device with a ~117 nm active layer (LQ-51/PCDTBT/PC_71_BM = 1:0.7:5) and two binary device with a ~60 nm active layer (LQ-51/PC_71_BM = 1:2.5) and a ~55 nm active layer (PCDTBT/PC_71_BM = 0.7:2.5), respectively. The two binary solar cells contain an approximately same quantity of individual components as in the ternary device. The absorption spectra of three blend films are as shown in Figure 4a, and the J–V characteristics of three devices are as shown in Figure 4b. The absorbance intensity of ternary film is almost the superposition of two binary films, which indicates that the ternary and two binary solar cells can be approximately regarded as the relationship between tandem solar cell and sub-cells. In addition, the J_SC_ of ternary solar cell (11.17 mA cm^−2^) are near the sum of that of two binary devices (6.63 mA cm^−2^ for LQ-51/PC_71_BM “sub-cell” and 5.08 mA cm^−2^ for PCDTBT/PC_71_BM “sub-cell”. In order to distinguish from sub-cells in tandem structure, the sub-cell here is in quotation marks), and the V_OC_ of ternary solar cell is located between that of two “sub-cells”, which corresponds to the concept of parallel-like structure. The EQE curves of ternary device and two “sub-cells” are as shown in Figure 4c. The EQE of the ternary device is at short wavelength range, where results mainly from PCDTBT and PC_71_BM responses, are almost the sum of those of two “sub-cells”. While at long wavelength range (700–800 nm), which is mainly attributed to LQ-51 response, the EQE of the ternary device is higher than the sum of those of two “sub-cells”. This might result from the enhanced hole mobility of ternary film.

**Figure 5:**
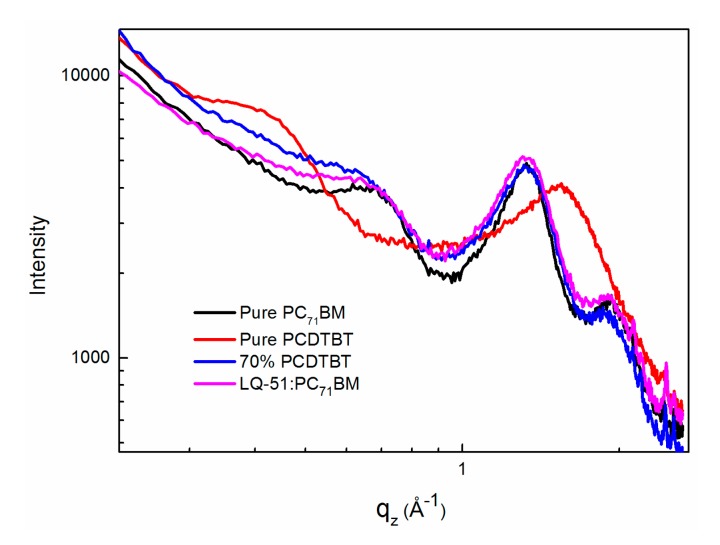
The out-of-plane grazing incidence X-ray diffraction (GIXRD) of pure PC_71_BM, pure PCDTBT, LQ-51/PC_71_BM binary film, and 70% PCDTBT-incorporated ternary film.

To understand the effect on the morphology of blend films by incorporating PCDTBT, we measured out-of-plane grazing incidence X-ray diffraction (GIXRD) of pure PC_71_BM, pure PCDTBT, LQ-51/PC_71_BM binary film, and 70% PCDTBT-incorporated ternary film (as shown in Figure 5). The pure PC_71_BM film shows three diffraction peaks at q = 0.7 Å^−1^, q = 1.33 Å^−1^, and q = 1.93 Å^−1^. The pure PCDTBT film shows lamellar (100) diffraction peak at q = 0.43 Å^−1^ and π–π stacking peak at q = 1.55 Å^−1^, while, there are only PC_71_BM stacking peaks in LQ-51/PC_71_BM binary film and 70% PCDTBT incorporated ternary film, which indicates that both LQ-51 and PCDTBT are amorphous in blend films. Also, the stacking peak of PC_71_BM at q = 0.7 Å^−1^ was extended in binary film, and becomes even broader when 70% PCDTBT is incorporated, which indicates that the PC_71_BM domain is smaller in ternary blend film.

**Figure 6:**
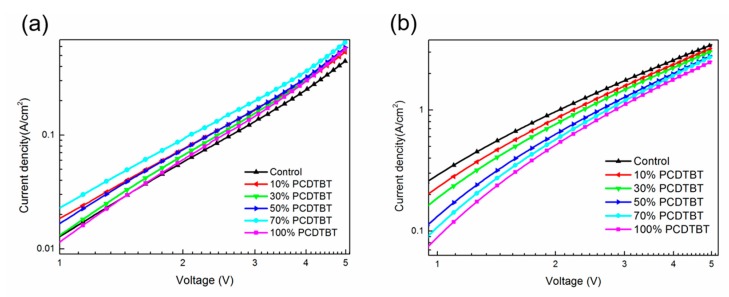
Dark J–V characteristics of hole-only (**a**) and electron-only (**b**) devices with different PCDTBT concentration.

As a donor material, PCDTBT shows much higher hole mobility (~1.84 × 10^−4^ cm^2^ V^−1^ s^−1^) compared with LQ-51 (~1.68 × 10^−5^ cm^2^ V^−1^ s^−1^), which may enhance the hole mobility of ternary films when it is incorporated into blend film. To study the charge carrier transport efficiency in ternary films, we measured the hole and electron mobility of blend films by space charge-limited current (SCLC) method. The device structures of hole-only and electron-only devices are ITO/MoO_3_/blend film/Au and ITO/PFN/blend film/Al, respectively. The dark J–V characteristics of hole-only and electron-only devices with different PCDTBT concentration are as shown in Figure 6. The hole mobility and electron mobility of blend films are as shown in Table 2. The hole mobility of blend films increases monotonously as the proportion of PCDTBT increasing up to 70% (from 1.18 × 10^−5^ cm^2^ V^−1^ s^−1^ to 3.84 × 10^−5^ cm^2^ V^−1^ s^−1^), and then decreases when the proportion is further up to 100%, which may be attributed to the disturbed hole transport pathway in LQ-51. While the electron mobility of blend films decreases monotonously as the proportion of PCDTBT increases (from 6.52 × 10^−5^ cm^2^ V^−1^ s^−1^ to 3.43 × 10^−5^ cm^2^ V^−1^ s^−1^), which results from the smaller PC_71_BM domain in ternary films and unefficient electron transport pathway. The reduced electron mobility is consistent with the measured results of the PC_71_BM responded EQE range. In addition, we notice that the hole and electron mobility become more balanced in the ternary blend films, which is beneficial to efficient charge extraction [24,25]. The μ_e_/μ_h_ decreases from 55.3 for LQ-51/PC_71_BM binary device to 9.8 for 70% PCDTBT incorporated ternary device, which is strongly related to the FF and PCE of solar cells.

**Figure 7:**
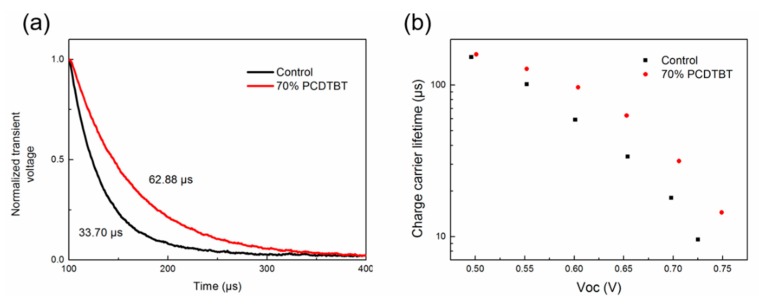
Transient photovoltage (TPV) measurement. (**a**) ΔV_OC_–time characteristics of binary and ternary devices when the V_OC_ of solar cells was kept at 0.65 V; (**b**) Charge carrier lifetime of binary and ternary devices measured under different V_OC_ conditions.

We further study the charge carrier characteristics in the binary and ternary device through transient photovoltage (TPV) measurements. The solar cells were exposed to a stabilized white light under open-circuit condition. When the V_OC_ of solar cells was kept at 0.65 V, the charge carrier lifetime was 33.70 μs and 62.88 μs for binary and ternary devices (as shown in Figure 7a), respectively. Then, we changed the distance between light source and solar cells to keep the device under different V_OC_ conditions. The charge carrier lifetime of the ternary device is much longer than that of the binary device under any V_OC_ condition (as shown in Figure 7b), which indicates less charge carrier recombination in the ternary solar cell. The incorporation of PCDTBT would add another hole transport pathway, which combined with high hole mobility of PCDTBT can significantly enhance charge extraction and reduce charge carrier recombination in ternary devices. Therefore, the FF and PCE increase significantly when 70% PCDTBT is incorporated.

## 4. Conclusions

In summary, we have successfully enhanced the performance of SQ derivative-based solar cells via incorporating a high hole mobility polymer with complementary absorption spectrum. We also demonstrate that the charge carrier transport in ternary blend film follows a parallel-like behavior. The holes transport in individual pathways without interacting with each other after exciton dissociation. The hole mobility and charge carrier lifetime increase resulted from the additional hole transport pathway. The parallel-like structure might be an elegant strategy to further enhancing the performance of other small molecule organic solar cells (SMOSCs) which are insensitive to processing treatments.

## Figures and Tables

**Table 1:** Photovoltaic parameters of binary and LQ-51/PCDTBT/PC_71_BM ternary solar cells, measured under AM 1.5 G solar illumination (100 mW cm^−2^). R_Sh_ and R_S_ extracted from illuminated J–V curves. D_1_ is LQ-51, D_2_ is PCDTBT. The PCE^ave^ is the average PCE and standard deviation calculated from 24 samples.

D_1_:D_2_:A	PCE (%)	J_SC_ (mA cm^−2^)	V_OC_ (V)	FF (%)	PCE^ave^ (%)	R_S_ (Ω cm^2^)	R_Sh_ (Ω cm^2^)
1:0:5	3.75	9.47	0.83	47.77	3.71 ± 0.12	11.27	321.54
1:0.1:5	4.04	9.74	0.84	49.43	4.04 ± 0.11	10.95	476.19
1:0.3:5	4.35	10.19	0.84	50.76	4.31 ± 0.07	9.31	512.82
1:0.5:5	4.44	10.12	0.85	51.64	4.46 ± 0.07	8.76	526.32
1:0.7:5	4.80	10.86	0.85	51.98	4.81 ± 0.12	8.59	555.56
1:1:5	4.64	10.46	0.87	50.92	4.64 ± 0.12	11.43	552.49
0:1:5	3.27	6.52	0.87	57.59	3.36 ± 0.13	11.18	1204.82

**Table 2:** The hole mobility (μ_h_) and electron mobility (μ_e_) of the binary and ternary blend films, evaluated using the Mott–Gurney law.

Blend Films	μ_h_ (cm^2^ V^−1^ s^−1^)	μ_e_ (cm^2^ V^−1^ s^−1^)	μ_e_/μ_h_
Control	1.18 × 10^−5^	6.52 × 10^−4^	55.3
10% PCDTBT	2.98 × 10^−5^	5.83 × 10^−4^	19.6
30% PCDTBT	2.65 × 10^−5^	5.14 × 10^−4^	19.4
50% PCDTBT	3.05 × 10^−5^	4.23 × 10^−4^	13.9
70% PCDTBT	3.84 × 10^−5^	3.76 × 10^−4^	9.8
100% PCDTBT	2.54 × 10^−5^	3.43 × 10^−4^	13.5
